# Effects of physical education, extracurricular sports activities, and leisure satisfaction on adolescent aggressive behavior: A latent growth modeling approach

**DOI:** 10.1371/journal.pone.0174674

**Published:** 2017-04-14

**Authors:** Sanghyun Park, Weisheng Chiu, Doyeon Won

**Affiliations:** 1 Department of Sport Industry Studies, Yonsei University, Seoul, Republic of Korea; 2 Department of Sport and Leisure Studies, Keimyung University, Daegu, Republic of Korea; 3 School of Sport Studies, Leisure and Nutrition, Liverpool John Moores University, Liverpool, United Kingdom; Johns Hopkins University Bloomberg School of Public Health, UNITED STATES

## Abstract

The present study aimed to investigate the longitudinal influence of physical education classes, extracurricular sports activities, and leisure satisfaction on aggressive behavior among South Korean adolescents. Data were drawn from the Korea Youth Panel Survey. We used latent growth curve modeling to explain the growth trajectory of adolescent aggressive behaviors and a multi-group analysis to investigate gender differences in aggressive behavior. The results indicated that adolescents’ aggressive behavior significantly changed with age. There were significant gender-based differences in the level of and changes in aggressive behavior over time. Both extracurricular sports activities and leisure satisfaction had significant influences on the changes in adolescents’ aggressive behavior with age, whereas physical education classes did not.

## Introduction

Aggression is one of the most prevalent and destructive behaviors to which adolescents may be exposed, and they are at a particular risk of finding themselves either the perpetrators of aggression or victims of bullying. The school may be the most common place where acts of aggression are perpetrated, as this is where a large proportion of adolescents spend much of their time [1]. Notably, school aggression in South Korea continues to be a serious issue, and the problem is becoming worse [2]. A panel survey conducted in 2012 by the Foundation for Preventing Youth Violence found that in a counseling center established in South Korea, 12% of students admitted to being bullied in school. A more serious problem was that approximately 45% of victimized students revealed that they felt suicidal because of school bullies [3]. In addition, the US Center for Disease Control reported that roughly 20% of students were bullied on school property and 16% of students were cyber-bullied [4]. This reveals that aggression in schools may cause fatal and irreparable damage in adolescents. Although schools have implemented a variety of violence prevention programs and curricula, violence in schools has not improved [5, 6]. Therefore, the question of how to curb violence in students has become a grave challenge for schools and educators [7].

Adolescent aggressive behavior may be influenced by various school-related factors [8]. Among those factors, physical education (PE) classes and extracurricular sports activities play a significant role in shaping adolescent behavior. Adolescents who participate in PE classes and sports activities with their peers may have more opportunities to develop self-regulation and the ability to control their aggressive behaviors [9]. In addition, leisure satisfaction can also be linked to low levels of antisocial behaviors such as burglary or bullying [10].

Numerous studies have investigated the aggressive behavior of adolescents in school [11], focusing on areas such as gender differences in aggressive behavior [8, 12, 13], interaction with peers [14–16], and role of parents and teachers [17]. However, to our knowledge, no study has investigated PE classes, extracurricular sports activities, leisure satisfaction, and aggressive behavior to determine whether and how these variables affect adolescents’ aggressive behavior. Furthermore, because of their cross-sectional designs, most previous studies failed to consider possible changes over the span of students’ time in school.

To gain a thorough understanding of the relationships between PE classes, extracurricular sports activities, leisure satisfaction, and aggressive behavior, the present study used a longitudinal design. Data were drawn from the Korea Youth Panel Survey (KYPS) to explore the longitudinal influences of PE classes, extracurricular sports activities, and leisure satisfaction on changes in adolescents’ aggressive behavior over 4 years. The findings of this study may offer insight and implications for school educators to help prevent school violence.

### Theoretical background and hypotheses formulation

#### Changes in aggressive behavior and gender differences

Aggression is a kind of impulsive behavior that tends to result in dangerous outbursts that may hurt people or destroy property [18]. The literature indicates that physical aggression is at its highest in early childhood and decreases with age because children are socialized away from physical aggression and learn to express their anger verbally; however, with age, verbal aggression also decreases [19, 20]. In most adolescents, overt physical and verbal aggression decreases during middle school because they develop more mature verbal and social-cognitive skills than in childhood [21, 22].

Over the past few decades, many studies have investigated gender differences in adolescents’ aggressive behavior. These studies showed a general tendency for males to be more overtly aggressive than females in adolescence, as females are socialized away from overt aggression more actively than males [12, 13, 19, 23, 24]. However, it should be noted that these patterns have not been examined among South Korean adolescents. Therefore, the present study established two hypotheses:

*H1a*: *South Korean adolescents’ aggressive behavior will change with age*.
*H1b*: *There will be gender differences in the changes in South Korean adolescents’ aggressive behavior*.

#### PE classes, extracurricular sports activities, and leisure satisfaction

Previous studies have suggested that PE classes and extracurricular sports activities play a crucial role in adolescent behavior. Through interaction with peers in PE classes or during extracurricular sports activities, adolescents may develop self-regulation skills and control their aggressive behavior [9]. Ratey and Hagerman reported that rebuilding sports facilities and extending the time of PE classes is a useful way to reduce aggressive behavior [25]. Previous studies on the influence of sports activities on adolescent behavior found that adolescents benefit from PE and after-school sports activities [26–30]. These findings suggest that PE classes and extracurricular sports activities have positive influences on adolescents’ social development and prosocial behaviors, including helping, sharing, donating, cooperating, volunteering, and showing consideration for others. Other studies have claimed that adolescents’ aggressive behavior may be reduced when they find gratification in their daily activities [31, 32]. Misra and McKean reported that adolescents’ aggressive behavior was reduced when they found satisfaction in their leisure activities [33]. Therefore, to test how PE classes, extracurricular activities, and leisure satisfaction affected South Korean adolescents’ aggressive behavior, we formulated three additional hypotheses:

*H2a*: *Participation in PE classes will influence the changes in South Korean adolescents’ aggressive behavior with age*.
*H2b*: *Participation in extracurricular sports activities will influence the changes in South Korean adolescents’ aggressive behavior with age*.
*H2c*: *Leisure satisfaction will influence the changes in South Korean adolescents’ aggressive behavior with age*.

## Materials and methods

### Ethics statement

This study followed the ethical guidelines set by the Yonsei University Institutional Review Board and the Yonsei College of Sciences in Education.

### Sample and data preparation

Participants in this study were selected from the KYPS conducted by the National Youth Policy Institute of South Korea from 2003 to 2006. The survey was implemented in 2003 using a stratified multi-stage cluster sampling method. A total of 3,449 adolescents (male: 1,725, female: 1,724) were selected to complete the survey every year up to 2008. The holding rate of the sample was over 90% in each year. Before data analysis, a data cleaning process was performed to improve data quality [34]. First, we eliminated 302 responses as the participants had been absent in two of the 4-year survey. Second, we removed 482 responses because those participants did not participate in a PE class in 2006. Finally, 18 responses were eliminated because of the existence of outliers (z-score outside ±3.29). Consequently, data for 2,647 respondents (male: 1,380, female: 1,267) were analyzed, which represented an appropriate number to reflect the sample’s tendencies in the whole population [35]. The demographic characteristics of the sample are shown in Table 1.

**Table 1:** Demographic variables.

Items		Frequency (n)	Proportion (%)
Gender	Male	1380	52.1
	Female	1267	47.9
Household income (monthly; KRW in thousand)	<100	240	9.1
	101–200	842	31.8
	201–300	765	28.9
	301–400	397	15.0
	>401	403	15.2
Private education expenses (monthly; KRW in thousand)	<10	707	26.7
	11–20	649	24.5
	21–30	621	23.4
	31–40	236	8.9
	>41	434	16.5
Father’s education level	Middle school	288	10.9
	High school	1186	44.8
	College	211	8.0
	University	776	29.3
	Graduate school	186	7.0
Mother’s education level	Middle school	455	17.2
	High school	1530	57.8
	College	156	5.9
	University	463	17.5
	Graduate school	43	1.6

### Measures

The survey instruments for this study included four scales: aggressive behavior, participation in PE classes, participation in extracurricular sports activities, and leisure satisfaction. The scales were adopted from the KYPS, as their validity and reliability had been previously confirmed. Aggressive behavior comprised six items evaluating the intentional use of physical aggression, including “If I am very angry, I will get into a physical fight”; “I hit back when someone hits me first”; “Compared with other people, I am more likely to get into a fight”; “When I get upset, I am impulsive and quick to throw stuff around”; “I cannot control my urges to beat up others”; and “I think I am like a walking bomb that could go off anytime.” PE class participation was assessed with the question, “How much did you participate in PE classes during the past year?” Leisure satisfaction was also assessed with one item, “Generally, I am satisfied with my leisure activities.” Aggressive behavior, participation in PE classes, and leisure satisfaction were measured on a five-point Likert scale. Participation in extracurricular sports activities was a dummy variable (see S1 Table). The group showing high involvement in extracurricular sports activities (e.g., soccer, basketball, body building, boxing, and tennis) was classified as “1,” and the group that mostly participated in other activities (e.g., watching TV, sleeping, and reading) was classified as “0.”

### Overview of latent growth curve model

The latent growth curve model can be explained as a combination of repeated ANOVAs with confirmatory factor analysis. It is an optical method to elucidate both inter-individual and intra-individual variations [36, 37]. Moreover, it presents the unique advantages of allowing the treatment of missing data and enabling us to conduct statistical analyses, even when the estimated durations between individuals are different [38]. The equation formula for the latent growth curve model is as follows.

$$Y_{ti}=\beta_{0i}+\beta_{1i}a_{t}+\varepsilon_{ti}$$

*Y*_*ti*_ represents the individual score observed on multiple occasions. *β*_0*i*_ represents the individual’s initial state (intercept), and *β*_1*i*_ represents the individual’s linear slope. The initial state and slope are latent factors serving as two pivotal components to clarify the individual growth curve. Furthermore, *a*_*t*_ represents the factor loading value, which helps to define the shape of change over time. Finally, *ε*_*ti*_ represents the residuals of the prediction on each occasion [39]. The latent growth curve model has a special characteristic in that it can divide two formulas based on two fixed parameters (*β*_0*i*_, *β*_1*i*_). This can be explained as:

$$\beta_{0i}=\gamma_{00}+\epsilon_{0i}$$

$$\beta_{1i}=\gamma_{10}+\epsilon_{1i}$$

Here, *γ*_00_ represents the grand mean of the initial status, and *γ*_10_ represents the mean of all the slopes of an individual. Thus, compared with conventional analytic methods such as repeated ANOVAs, which stipulate strict statistical preconditions, the latent growth curve model offers great flexibility for estimating parameters and interpreting results, as its mathematical formula can be separated into an entire mean and an individual measurement error.

**Fig 1:**
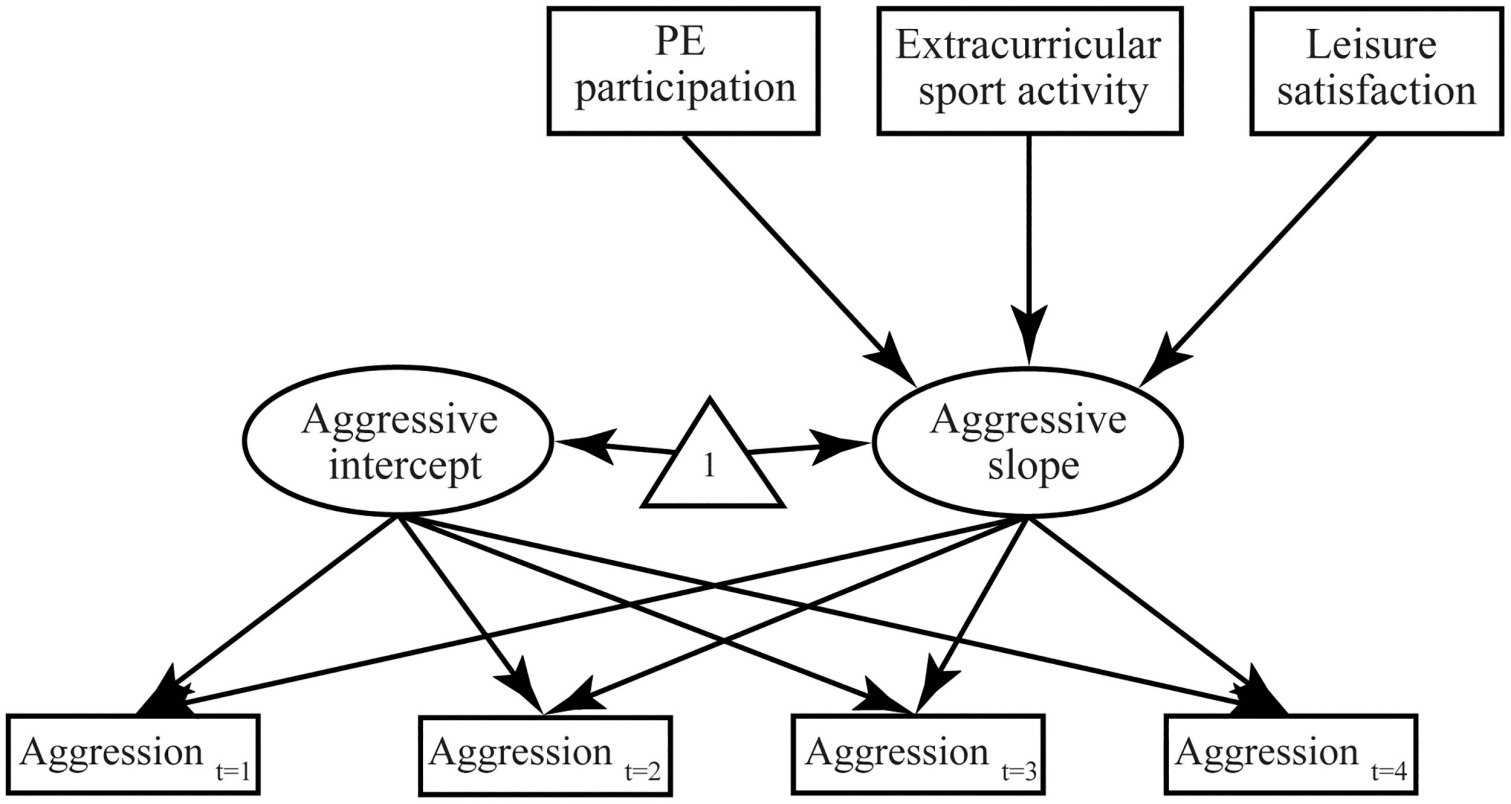
Research model.

The latent growth curve model illustrated in Fig 1 was used in this study. We used a triangle [40] to visualize the means (intercepts) of the estimated latent variables.

### Missing data procedure

As a next step, we reviewed the remaining data and found some missing values in 142 of 2,647 surveys (5.36%). The missing values indicate the instance that data values were not present for the variable in question. Although missing values are fairly common in longitudinal data, they might have a significant influence on the results. To manage missing data, some scholars adopt either a “complete case analysis method” or an “available case analysis method.” However, these methods are not recommended as they create biased samples [41]. Thus, a dummy coding was used for each variable in turn, scoring 0 for non-missing data and 1 for missing data, to investigate whether the dummy variable is related to other variables of interest [42]. The results indicated that missingness was related to other variables (*p*_s_ > .08), indicating a pattern of missing at random (MAR). With an assumption of MAR, we used full-information maximum likelihood (FIML) as FIML is an efficient and unbiased method of estimating parameters. FIML is also a useful method for estimating stable parameters [43, 44].

### Data analysis

Firstly, the latent growth curve model was analyzed to elucidate the growth trajectory of adolescent aggressive behavior. Secondly, a multi-group analysis was performed to identify the gender difference in the growth trajectory of aggressive behavior. Finally, we analyzed the research model to test the hypotheses. These processes were performed with the Mplus 7.0 software. A chi-square statistic index, comparative fit index (CFI), Tucker and Lewis index (TLI), and root mean square error of approximation (RMSEA) were used to test the adequacy of the research model. According to the criteria suggested by Hu and Bentler [45], CFI and TLI values should be larger than .90, and the RMSEA value should be below .08.

## Results

### Descriptive statistics

The means, standard deviations, skewness, kurtosis, and correlation coefficients of the studied variables are presented in Table 2. According to the criteria for skewness and kurtosis, all variables satisfied the assumption of a normal distribution. Moreover, the correlation coefficients for all variables were under .80, indicating no restriction from multicollinearity.

**Table 2:** Descriptive statistics and pearson’s correlation matrix.

	1	2	3	4	5	6	7
1^st^ aggression	1						
2^nd^ aggression	.474***	1					
3^rd^ aggression	.448***	.459***	1				
4^th^ aggression	.382***	.409***	.516***	1			
PE participation (dummy)	.015	.047*	.041*	.024	1		
Extracurricular sports activities	-.005	.013	.050*	.039*	.246***	1	
Leisure satisfaction	-.101	-.112	-.112***	-.124***	.119***	.129***	1
Mean	2.751	2.767	2.696	2.672	3.301	.341	3.2996
*SD*	.700	.713	.692	.683	.759	.474	.574
Skewness	.066	.004	.110	.056	-.014	.671	-.083
Kurtosis	3.145	3.212	3.415	3.186	-3.195	-4.552	3.156

*p < .05,

***p < .001

### Growth trajectory of aggression

To determine the best-fitting model for the change in adolescents’ aggressive behavior, we examined and compared the unconditional, linear, and quadratic models. Although all models showed appropriate fit indices, the linear model showed the best model fit (Table 3). Therefore, we concluded that adolescents’ aggression decreased in a linear manner, and the initial status and slope of aggressive behavior differed between adolescents as their variances were significant. Moreover, the correlation coefficient between the initial status and the slope was −.401. This means that adolescents who showed low aggression in the initial period had a steeper slope than adolescents who showed high aggression in the initial period. Therefore, H1a was supported.

**Table 3:** Aggression model.

Model	χ^2^	*df*	TLI	CFI	RMSEA	
Quadratic model	8.77	1	.969	.997	.054	
Linear model	17.29	5	.990	.995	.030	
Unconditionalmodel	127.29	8	.940	.952	.075	
Model	Intercept		Linear slope		Quadratic slope	
	Mean	Variance	Mean	Variance	Mean	Variance
Quadratic model	2.758***	.238***	-.001	.004	-.009*	.003
Linear model	2.765***	.269***	-.030***	.019***		
Unconditional model	2.758***	.238***				

*p < .05,

***p < .001

Note: the correlation coefficient of the linear model was −.401***

### Gender differences

Next, we performed a multi-group analysis to examine gender-based differences. Although configural invariance and metric invariance (factorial invariance or weak invariance) are prerequisites for examining group differences [46], only configural invariance was a prerequisite in the present LGM, as the factor coefficient and intercept were already fixed. The configural invariance test showed the male and female models were appropriate for performing multi-group analyses. In comparison with the metric invariance model (baseline model), the intercept invariance model, Δχ^2^ (1) = 20.18 > 3.84, and the slope invariance model, Δχ^2^(1) = 28.87 > 3.84, were not acceptable, as the differential chi-square value of the two models exceeded the critical value of 3.84 (Table 4). This indicates that the initial aggression status of females was significantly higher than that of males. The slope for females was also significantly steeper than that for males, supporting H1b.

**Table 4:** Gender differences in aggression.

Model	χ^2^	*df*	TLI	CFI	RMSEA	Initial status		Slope	
						Estimate	SE	Estimate	SE
Male	12.471	5	.986	.993	.033	2.710***	.017	-.005	.007
Female	11.088	5	.992	.996	.031	2.825***	.019	-.057***	.007
Baseline model	23.55	10	.989	.995	.023				
Intercept invariance	43.73	11	.976	.987	.034				
Slope invariance	52.42	11	.970	.984	.038				

***p < .001

**Fig 2:**
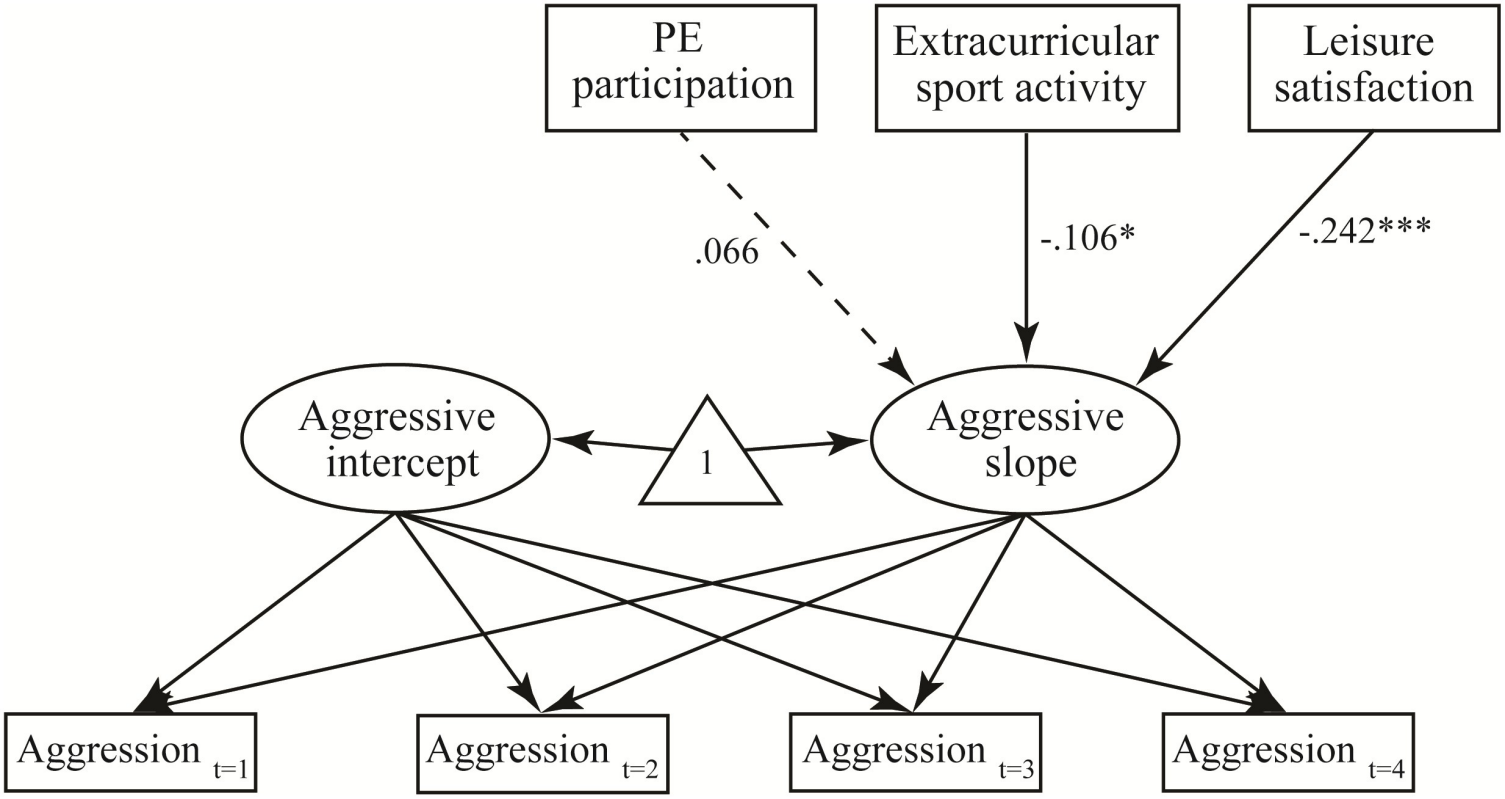
Path analysis results. *p < .05, ***p < .001

**Fig 3:**
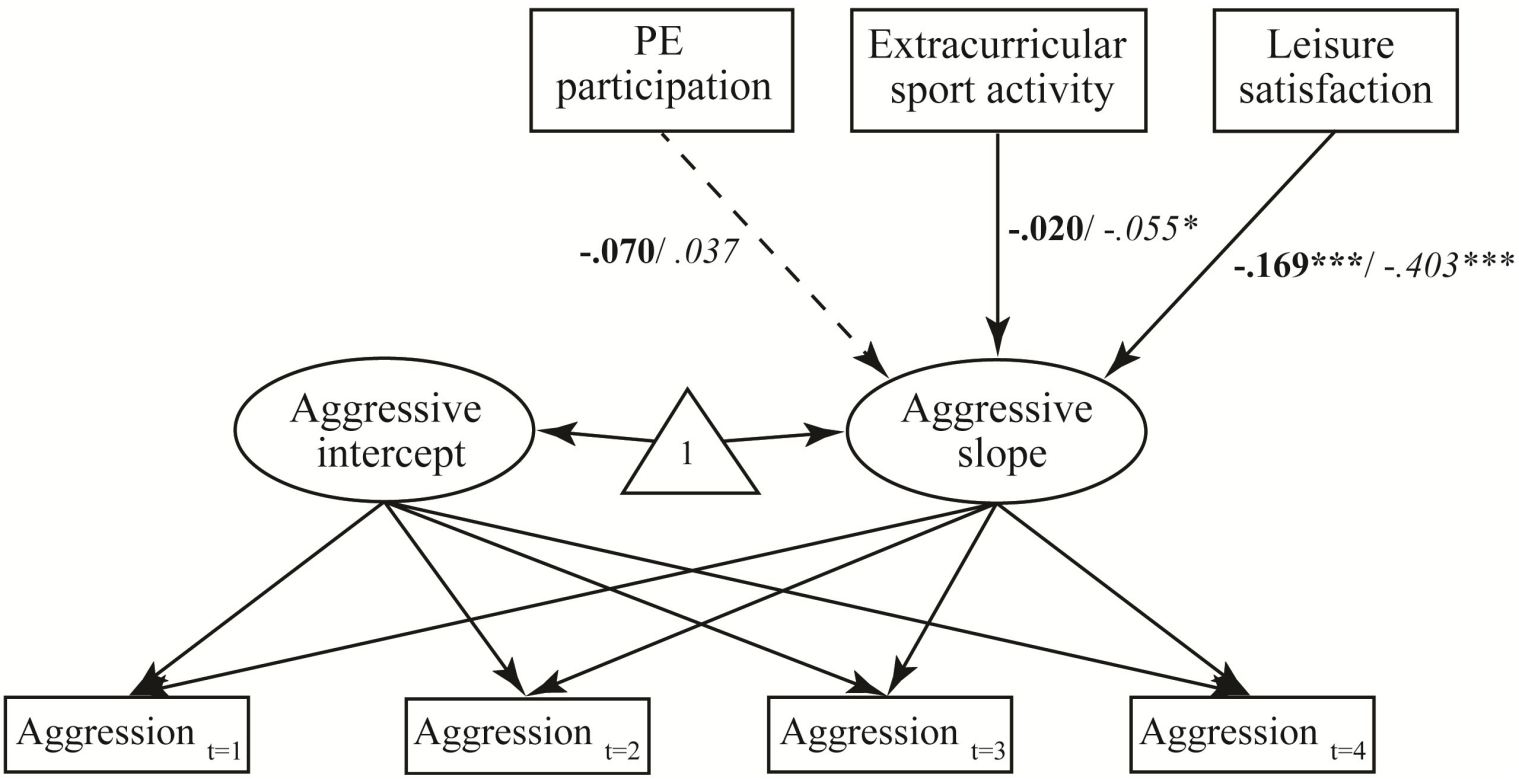
Path coefficients for males (bold) and females (italics). *p < .05, ***p < .001

As shown in Fig 2 and Table 5, we tested three hypotheses with the research model. The effect of PE participation on the slope of aggressive behavior was insignificant (β = .005, *t* = 1.333, *p* = .183), indicating that H2a was rejected. However, participation in extracurricular sports activities had a significant effect on the aggression slope (β = −.106, *t* = −2.185, *p* = .029). Leisure satisfaction also had a significant effect on the aggression slope (β = −.242, t = −5.022, *p* < .000). Therefore, H2b and H2c were confirmed. In addition, we performed a multiple group analysis to assess the difference between males and females. Female adolescents’ participation in extracurricular sports activities had a significant effect on the aggression slope (Fig 3).

**Table 5:** Hypotheses testing.

Hypothesis	B	S.E	β	t	p	Decision
H2a: PE participation to the slope of aggression	.007	.066	.005	1.333	.183	Reject
H2b: extracurricular sports activity to the slope of aggression	-.024	.011	-.106	-2.185	.029	Accept
H2c: leisure satisfaction to the slope of aggression	-.035	.007	-.242	-5.022	.000	Accept

## Discussion

### Aggression pattern and gender differences

The most appropriate model for explaining adolescent aggression was the linear model. Specifically, the coefficient of the linear slope was −.030, meaning that adolescents’ aggression decreased over time. Moreover, there was a significant discrepancy between male and female adolescents in terms of initial status and slope. Specifically, the females’ initial aggression status was higher than that of males, whereas the slope for males was steeper than for females. These results are consistent with previous studies [8, 12, 13, 19, 23, 24]. In these studies, female students showed higher levels of anti-social behavior and aggression than males because female students were inclined to internalize aggression. In contrast, some studies found that male aggression was much higher than female aggression [47]. This difference may be attributed to the heterogeneity of the instruments used to measure aggression. Many studies assessed aggression using overt behaviors, such as smoking, drinking, and anti-social behavior. However, these studies did not consider psychological and internal aggression.

In the context of South Korean male adolescents, it has been suggested that certain aggressive behaviors towards their peers were ways to express their manhood. As such, they were to some extent open-minded about aggressive behaviors. In contrast, female adolescents are expected to be feminine, and the society’s stigmatization of aggression is much higher for women than for men. Moreover, Belknap explained this result by noting that female students are strictly monitored by parents, schools, and society, and their aggressive behavior is therefore repressed [48]. However, internalized aggression is an essential factor in the emergence of external aggressive behavior and potentially triggers various anti-social behaviors. Currently, urgent efforts to reduce aggression through educational programs and management are needed, particularly as the problems of female aggression and anti-social behavior are increasing rapidly.

### Effect of PE participation

We found that PE participation had no impact on the slope of aggressive behavior. This was inconsistent with previous studies [26]. Although PE in school may have a positive influence on adolescents’ behavior, the potential negative effects of PE cannot be ignored [49]. Sports activities can teach individuals to follow social rules and carry high responsibilities, but they also may create stress and aggressiveness. Our findings showed that PE participation did not have a significant positive effect on the aggression slope. Many previous studies suggested that sports programs should be enhanced as they may have a positive impact on optimism and life satisfaction (Koo & Lee, 2014). Sports activities in school were also found to play a fundamental role in social development and social integration [50, 51]. In addition, PE in school was considered an effective method to prevent and cure anti-social behavior. Despite these advantages, the impact of PE classes in South Korea might be different and need further investigation. Most South Korean adolescents experience severe academic pressure and prefer to focus on core subjects such as Mathematics and English. PE is regarded as a minor subject and is not widely encouraged in middle and high school. PE hours may even be used for core subjects or academic study time [52]. These factors reduce the beneficial outcomes of PE. Moreover, in South Korea, the design of many PE classes is monotonous. Many PE teachers typically teach the same activities, such as soccer or basketball, to students across all years [52]. Therefore, in this context, participation in PE classes has a limited positive influence on adolescents in South Korea.

### Effect of participation in extracurricular sports activities

Unlike PE classes in school, extracurricular sports activities showed a significant and negative impact on the aggression slope. Extracurricular sports activities also played a more significant role in decreasing the aggression slope for female adolescents. Extracurricular sports activities are a known outlet for psychological stress stemming from unstable relationships and anxiety [27, 53, 54]. As such, they can serve as a mitigating factor for aggressive behavior. Specifically, extracurricular sports activities promote both physical advantages (e.g., health preservation and stamina improvement) and psychological advantages by encouraging self-effacement, mutual respect, and consideration. Ultimately, these benefits may contribute to encouraging more ethical and less delinquent behavior in adolescents. To reduce adolescents’ aggressive behavior, their participation in various extracurricular sports activities should be considered by school educators and administrators. In addition, the differences between male and female adolescents should be taken into account.

### Effect of leisure satisfaction

Our findings showed that leisure satisfaction had a significant effect in terms of a lower aggression slope. This is consistent with previous studies [31–33], which argued that satisfaction and self-esteem gained from leisure activities could have a positive impact on adolescents’ lives. That is, adolescents who are satisfied with their leisure time are likely to have a lower potential for aggressive behavior. This also suggests that increased opportunities to participate in leisure activities play a critical role in satisfaction. As noted, many adolescents in South Korea are under significant academic pressure and may therefore feel confused about the future. To overcome this difficulty, the government and schools should provide ample opportunities for adolescents to participate in and enjoy leisure activities. The 5-day school week system may be a useful way to reduce South Korean adolescents’ aggression and to provide more chances to participate in weekend leisure activities.

## Conclusion

Using an LGM approach and secondary data from the KYPS, the present study clearly demonstrated the benefits of extracurricular sports activities and leisure satisfaction on adolescents’ aggressive behavior. Our findings showed that adolescents’ aggressive behavior decreased over time and that the speed of change was significantly higher in females than in males. Moreover, extracurricular sports activities and leisure satisfaction had a positive influence on this aggression change. Our results could provide critical implications for school educators and may help them develop new sports programs.

Despite these insightful implications, our study has several limitations. First, it only used three independent variables: participation in PE classes, participation in extracurricular sports activities, and leisure satisfaction. However, there may be many other factors influencing adolescents’ aggressive behavior, such as their parents, peer groups, teachers, and the school environment. These variables should be considered in future research. Second, participation in PE classes and leisure satisfaction were treated as invariant variables because of the lack of theoretical background on change. Therefore, this study was limited in understanding the unique effects of the participation in PE classes and leisure satisfaction at each point in time. Future studies should use an autoregressive model or a cross-lagged model to identify more precise causal relationships.

## Supporting information

S1 Table The survey items. (DOCX)
