# Correction: Effects of physical education, extracurricular sports activities, and leisure satisfaction on adolescent aggressive behavior: A latent growth modeling approach

**DOI:** 10.1371/journal.pone.0251221

**Published:** 2021-04-29

**Authors:** Sanghyun Park, Weisheng Chiu, Doyeon Won

In the ‘Aggression pattern and gender differences’ subsection of the Discussion, there is an error in the fourth sentence of the first paragraph. The correct sentence is: Specifically, the females’ initial aggression status was higher than that of males, whereas the slope for males was less steep than for females.
