# Supplementary material for: Effects of physical education, extracurricular sports activities, and leisure satisfaction on adolescent aggressive behavior: A latent growth modeling approach
Source: PLoS One. 2017 Apr 14;12(4):e0174674. doi: 10.1371/journal.pone.0174674 (PMC5391921; doi:10.1371/journal.pone.0174674)

**Fig 1. Research model.**


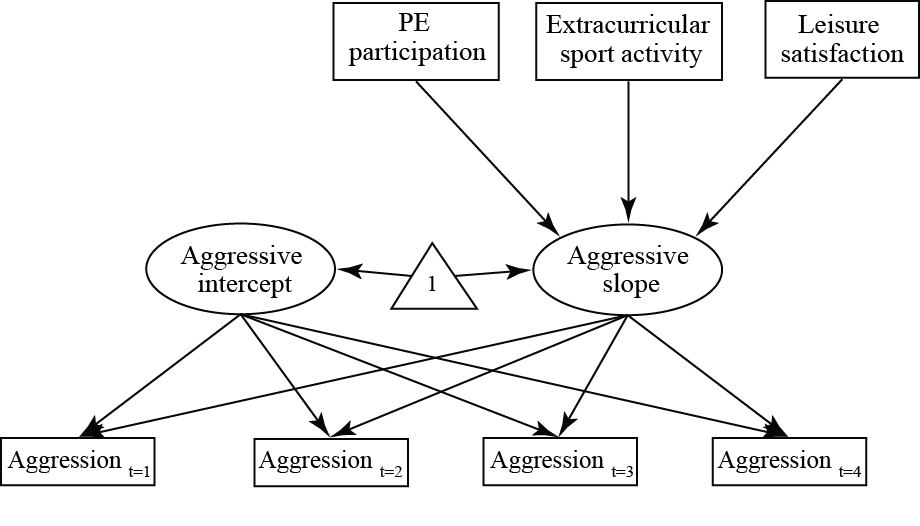


**Fig 2. Path analysis results.** **p < .*05, ****p < .*001


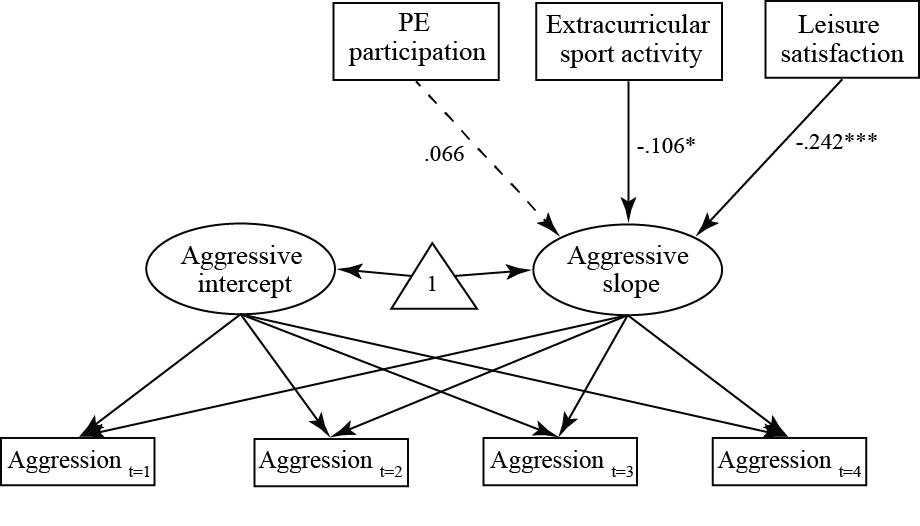


**Fig 3. Path coefficients for males (bold) and females (italics).** **p < .*05, ****p < .*001


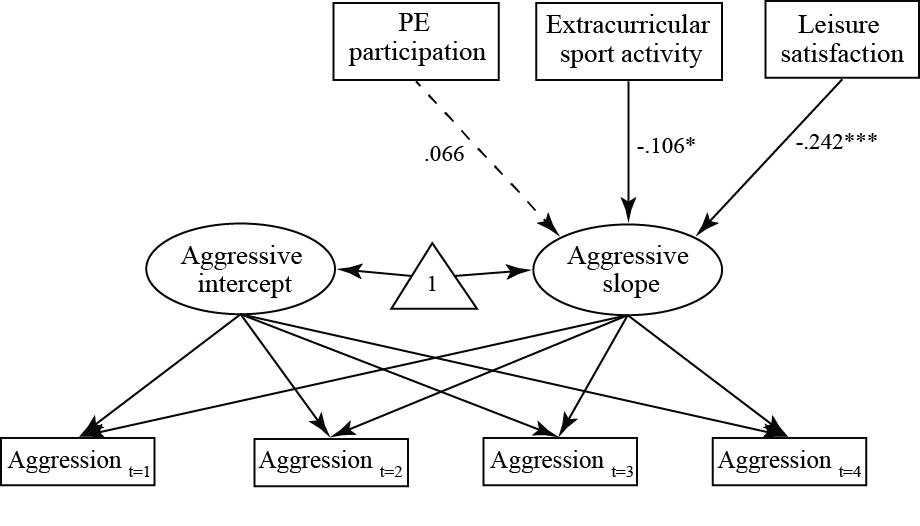

Supplement: S1 Table — (DOCX) [file pone.0174674.s001.docx]
